# Pyneal: Open Source Real-Time fMRI Software

**DOI:** 10.3389/fnins.2020.00900

**Published:** 2020-09-15

**Authors:** Jeff J. MacInnes, R. Alison Adcock, Andrea Stocco, Chantel S. Prat, Rajesh P. N. Rao, Kathryn C. Dickerson

**Affiliations:** ^1^Institute for Learning and Brain Sciences, University of Washington, Seattle, WA, United States; ^2^Department of Psychiatry and Behavioral Sciences, Center for Cognitive Neuroscience, Duke Institute for Brain Sciences, Duke University, Durham, NC, United States; ^3^Department of Psychology, University of Washington, Seattle, WA, United States; ^4^Department of Computer Science and Engineering, Center for Neurotechnology, University of Washington, Seattle, WA, United States

**Keywords:** real-time, functional magnetic resonance imaging, neurofeedback, open source software, python (programming language), neuroimaging methods, rt-fMRI

## Abstract

Increasingly, neuroimaging researchers are exploring the use of real-time functional magnetic resonance imaging (rt-fMRI) as a way to access a participant’s ongoing brain function throughout a scan. This approach presents novel and exciting experimental applications ranging from monitoring data quality in real time, to delivering neurofeedback from a region of interest, to dynamically controlling experimental flow, or interfacing with remote devices. Yet, for those interested in adopting this method, the existing software options are few and limited in application. This presents a barrier for new users, as well as hinders existing users from refining techniques and methods. Here we introduce a free, open-source rt-fMRI package, the Pyneal toolkit, designed to address this limitation. The Pyneal toolkit is python-based software that offers a flexible and user friendly framework for rt-fMRI, is compatible with all three major scanner manufacturers (GE, Siemens, Phillips), and, critically, allows fully customized analysis pipelines. In this article, we provide a detailed overview of the architecture, describe how to set up and run the Pyneal toolkit during an experimental session, offer tutorials with scan data that demonstrate how data flows through the Pyneal toolkit with example analyses, and highlight the advantages that the Pyneal toolkit offers to the neuroimaging community.

## Introduction

Real-time functional magnetic resonance imaging (rt-fMRI) is an emerging technique that expands the scope of research questions beyond what traditional neuroimaging methods can offer (Sulzer et al., 2013a; Stoeckel et al., 2014; Sitaram et al., 2017; MacInnes and Dickerson, 2018). With traditional fMRI, brain activation is measured concurrently but independently from the experiment. All analyses (e.g., correlating behavior or cognitive state with brain activations) therefore, take place after the scan^1^ is completed, once the brain images and behavioral data have been saved and transferred to a shared location. In contrast, *real-time fMRI* is an approach whereby MRI data is accessed and analyzed throughout an ongoing scan, and can be incorporated directly into the experiment. Technological advances over the last decade have made it feasible to reconfigure an MRI environment to allow researchers to access and analyze incoming data at a rate that matches data acquisition. A few key advantages that rt-fMRI provides over traditional fMRI include the ability to: (1) monitor data quality in real time, thereby saving time and money, (2) provide participants with feedback from a region or network of regions in cognitive training paradigms, and (3) use ongoing brain activation as an independent variable to dynamically control the flow of an experimental task.

While rt-fMRI has risen in popularity over the past decade (Sulzer et al., 2013a), the majority of imaging centers around the world remain unequipped to support this technique. In the past, this was primarily due to the computational demands exceeding scanner hardware capabilities [e.g., reconstructing and analyzing datasets composed of >100 k voxels at a rate that matched data acquisition was not feasible (Voyvodic, 1999)]. Excitingly, modern day scanners available from each of the major MRI manufacturers – GE, Philips, and Siemens – are now outfitted with multicore processors, capable of operating in parallel to reconstruct imaging data and write files to disk while a scan is ongoing.

The availability of fMRI data in real time presents novel opportunities to design experiments that incorporate information about ongoing brain activation. However, finding the right software tool to read images across multiple data formats, support flexible analyses, and integrate the results into an ongoing experimental presentation is a challenge. To date, the existing software options are limited for one or more reasons, including: cost (requiring a commercial license or dependent upon commercially licensed software such as Matlab) or a constrained choice of analysis options [e.g., region of interest (ROI) analysis only].

In this article, we describe the Pyneal toolkit, an open source and freely available software package that was developed to address these limitations and support real-time fMRI. It is written entirely in Python, a programming language that offers flexibility and performance, balanced with readability and widespread support among the neuroimaging community. The Pyneal toolkit was built using a modular architecture to support a variety of different data formats, including those used across all three major MRI scanner manufacturers – GE, Philips, and Siemens. It offers built-in routines for basic data quality measures and single ROI summary statistics, as well as a web-based dashboard for monitoring the progress of ongoing scans. Its primary advantage, however, is that it offers an easy-to-use scaffolding on which users can design fully customized analyses to meet their unique experimental needs (e.g., neurofeedback from multiple ROIs, dynamic experimental control, classification of brain states, brain-computer interaction). This flexibility allows researchers full control over which neural regions to include, which analyses to carry out, and how the results of those analyses may be incorporated into the overall experimental flow. Moreover, computational and technological advances have ushered in new and more sensitive approaches to fMRI analyses. As the field continues to evolve, the ability to customize analyses within the Pyneal toolkit will allow researchers to quickly adapt new analytic methods to real-time experiments.

The Pyneal toolkit was designed to offer a powerful and flexible tool to existing rt-fMRI practitioners as well as to lower the burden of entry for new researchers or imaging centers looking to add this capability to their facilities. Here we provide an overview of the software architecture, describe how it is used, offer tutorial data and analyses demonstrating how to use the Pyneal toolkit, and discuss the advantages of the Pyneal toolkit. We conclude by describing both limitations of and future directions for the Pyneal toolkit.

## Method

The Pyneal toolkit is available at https://github.com/jeffmacinnes/pyneal and full documentation is online at https://jeffmacinnes.github.io/pyneal-docs/.

### Overview

The Pyneal toolkit was created as a flexible and open-source option for researchers interested in pursuing real-time fMRI methods. The entire codebase is written in Python 3^2^ and integrates commonly used neuroimaging libraries (e.g., Nipy, NiBabel). For users developing customized real-time analyses, Python has a low burden of entry (compared to languages like Java or C++), while at the same time offers performance measures that meet or exceed the needs of basic research applications, in part due to backend numeric computing libraries (e.g., Numpy, Scipy) that are wrapped on top of a fast, C-based architecture.

**FIGURE 1:**
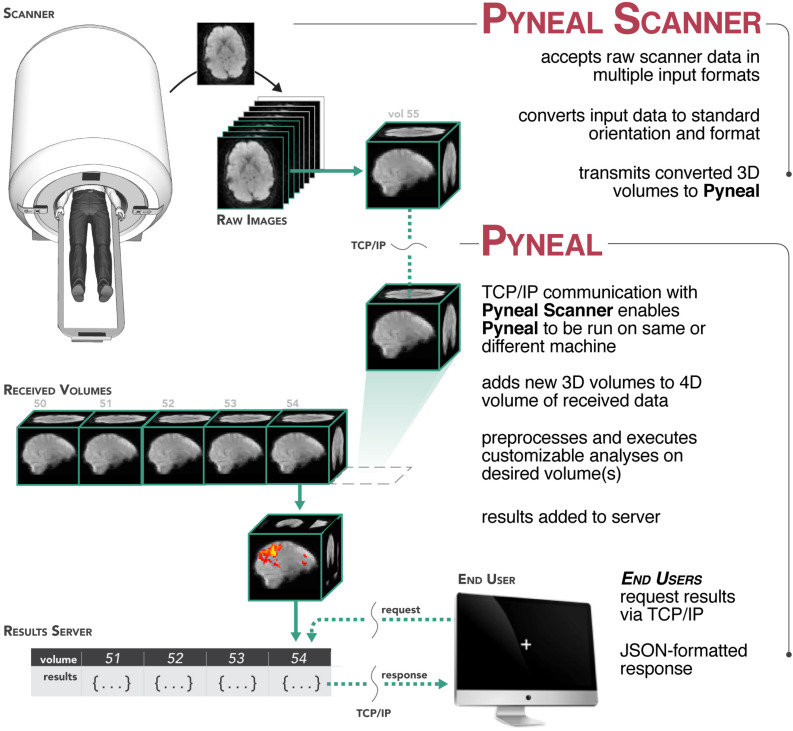
Overview of the Pyneal toolkit. The Pyneal toolkit consists of two modules: Pyneal Scanner and Pyneal. Pyneal Scanner receives the raw data and transforms it into a standardized format for Pyneal to use. Pyneal analyzes the data in real time and makes it available for subsequent use (e.g., by a remote End User for experimental display). Pyneal Scanner and Pyneal can operate on the same computer (e.g., dedicated analysis computer) or separate computers (as required by the specific scanning environment).

In order to support a wide range of data types and computing environments, the software is divided into two primary components: Pyneal Scanner and Pyneal^3^ (see Figure 1). The two components communicate via TCP/IP connections, allowing users the flexibility to run the components on the same or different machines as required by their individual scanning environments^4^. Internally, Pyneal uses ZeroMQ^5^, a performant and reliable messaging framework, for all TCP/IP-based communication among its core processes.

**FIGURE 2:**
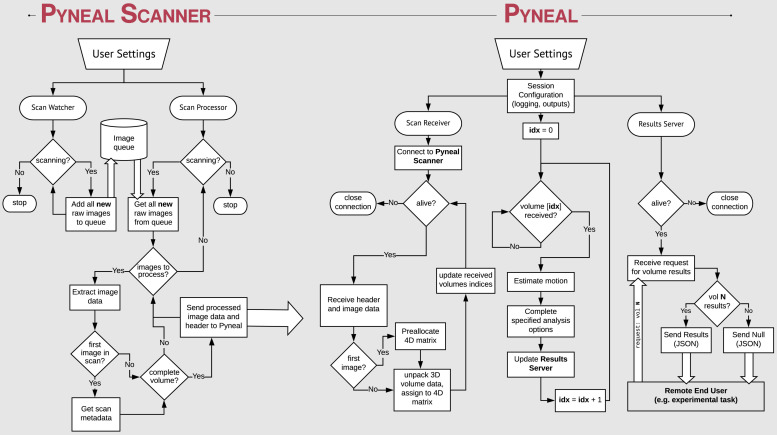
Process flow diagram illustrating the multi-threaded nature of the Pyneal toolkit. Pyneal Scanner has two sub-modules: a scan watcher and scan processor. The scan watcher monitors and adds all new raw images to a queue. The scan processor receives all new raw images from the queue, extracts the image data, transforms it to a standardized format, and sends it to Pyneal for analysis. Pyneal operates as an independent, multi-threaded component and has three sub-modules: scan receiver, scan processor, and results server. The scan receiver receives formatted data from Pyneal Scanner and sends it to the scan processor, which completes the specified analyses and sends them to the results server. The results server listens to incoming requests from End Users (e.g., experimental task).

During a scan, Pyneal Scanner is responsible for converting data into a standardized format and passing it along to Pyneal (see Figure 2). Pyneal receives incoming data, carries out the specified preprocessing and analysis steps, and stores the results of the analysis on a locally running server. Throughout the scan, any remote End User (e.g., a workstation running the experimental task) can retrieve analysis results from Pyneal at any point. Each of these components is discussed in greater detail below.

### Pyneal Scanner

Given the range of potential input data formats, depending on the scanning environment, we aimed to standardize the incoming data in a way that allows subsequent processing steps to be environment agnostic. Thus we divided the overall Pyneal toolkit architecture into two components that operate independently, enabling one component, Pyneal Scanner, to adapt to the idiosyncrasies of the local scanning environment without affecting the downstream processing and analysis stages of the Pyneal component (see Figure 2).

Architecturally, Pyneal Scanner uses a multithreaded design with one thread *monitoring* for the appearance of new image data, and a second thread *processing* image data as it appears. This design allows Pyneal Scanner to efficiently process incoming scan data with minimal latency (in practice, under typical scanning conditions, the latency between when new image data arrives and is processed is on the order of tens of milliseconds). Throughout a scan, new images that appear from the scanner are placed into a queue. The processing thread pulls individual files from that queue and converts the data to a standardized format. In addition, header information from the first images to arrive is processed to determine key metadata about the current scan, including total volume dimensions, voxel spacing, total number of expected time points, and the affine transformation needed to reorient the data to RAS+ format (axes increase from left to right, posterior to anterior, inferior to superior).

Pyneal Scanner is initialized through a simple configuration text file specifying the scanner type and paths to where data files are expected to appear throughout a scan. Users can create this file manually, or follow the command line prompts when first launching; in either case, once Pyneal Scanner is configured at the start of a session, it does not need to be modified, unless the scanning environment itself is modified. In that case, users can update Pyneal Scanner without having to add any additional modifications to downstream processes in Pyneal. Regardless of how and where the data arrives from the scanner, as long as Pyneal Scanner continues to output data in the expected format, subsequent stages in the pipeline will proceed unaffected. This is a significant advantage that provides researchers the necessary latitude to customize the installation to their unique environment.

Pyneal Scanner has built-in routines for handling common data formats used in GE (e.g., 2D dicom slice files), Siemens (e.g., 3D dicom mosaic files), and Philips scanners (e.g., PAR/REC files), and is easily extensible to incorporate additional formats that may emerge in the future.

As soon as a complete volume (i.e., 3D array of voxel values from a single time point) has arrived, it is passed along to Pyneal via a dedicated TCP/IP socket interface. This arrangement allows Pyneal Scanner and Pyneal to run on separate machines or as separate processes on the same machine, depending on the particular requirements of the local scanning environment. For instance, if newly arriving images are only accessible from the scanner console itself, Pyneal Scanner can run on that machine, monitoring the local directory where new images appear, and then transferring processed volumes to Pyneal running on a separate dedicated machine. Alternatively, the scanner network configuration may be such that it is possible to remotely mount the directory where new images appear, allowing Pyneal Scanner and Pyneal to run concurrently on the same machine.

Each transmitted volume from Pyneal Scanner to Pyneal occurs in two waves: First, Pyneal Scanner sends a JSON-formatted header that contains relevant metadata about the current volume, including the time point index and volume dimensions. Second, it sends the numeric array representing the volume data itself. Pyneal uses the information from the header to reconstruct the incoming array, store it as a memory- and computation-efficient Numpy array, and index the volume in a way to facilitate subsequent processing and analysis steps.

### Pyneal

Pyneal is divided up into three distinct submodules that operate efficiently in a multithreaded configuration: submodule 1: the scan receiver, accepts incoming data from Pyneal Scanner; submodule 2: the processing module, oversees the preprocessing and analysis stages on each incoming volume, and submodule 3: the results server fields requests for data from remote End Users throughout the scan (see Figure 2).

As described above, throughout a scan Pyneal’s submodule 1 (scan receiver) receives re-formatted data from Pyneal Scanner. Each new data point is represented as a 3D matrix of voxel values corresponding to a single sample (i.e., one TR). The JSON header that Pyneal Scanner provides with every transmission allows Pyneal to reconstruct the 3D volume with the correct dimensions, as well as assign it the proper index location in time. Each new volume is passed to the proper location of a preallocated 4D matrix that incrementally fills in throughout the scan.

Submodule 2 (processing module) accepts each 3D volume and submits it through preprocessing and analysis stages. The preprocessing stage estimates motion using a histogram registration algorithm and yields mean displacement in millimeters relative to a fixed reference volume from the start of the run (absolute motion), as well as relative to the previous time point (relative motion) (Jenkinson, 2000).

The analysis stage takes the preprocessed volume and runs the specified analyses or computations on the volume. Users have the option of selecting from built-in analysis routines (including calculating a weighted or unweighted mean signal within a supplied ROI mask), or, importantly, can generate and include their own custom analysis script (written in Python) that will be executed on each volume. The ability to design and execute customized analyses in real-time provides researchers the freedom to measure and use ongoing brain activations however they desire. See *Using Pyneal* below for more details on selecting an analysis or building a custom analysis script.

The analysis stage is capable of computing and returning multiple results on each volume (e.g., mean signal from multiple distinct ROIs). The computed results are tagged with the corresponding volume index, and passed along to the third submodule: the results server.

Submodule 3, (the results server), listens for and responds to incoming requests for specific results from an End User throughout the scan. An End User is anything that may wish to access real-time results throughout an on-going scan (e.g., experimental presentation software that will present results as neurofeedback to the participant in the scanner). To request results, the End User sends a specific volume index to the results server via a TCP/IP socket interface. The result server receives the request and checks to see if the requested volume has arrived and been analyzed. Responses are sent as a JSON-formatted reply to the End User. If the requested volume has not been processed yet, the reply message from the Result Server will contain the entry foundResults: False; if the requested volume exists, the Results Server retrieves the requested results for that volume, and sends a reply message to the End User that contains foundResults: True as well as the full set of results for that volume. The End User can then parse and make use of the results as needed (e.g., update a graphical display showing mean percent signal change in an ROI).

At the completion of each run, Pyneal creates a unique output directory for the current scan. The scan data is written to this directory as a 4D NIFTI image, along with a JSON file containing all computed results as well as log files.

### Using Pyneal

Once installed, users can interact with and customize Pyneal via configuration files and graphical user interfaces (GUIs). At the start of a new scan, the user needs to launch both Pyneal Scanner and Pyneal.

Launching Pyneal Scanner is done via the command line. Pyneal Scanner uses a configuration text file to obtain parameters specific to the current computing environment, including the scanner make and the directory path where new incoming data is expected to appear (see example in the Full Pipeline tutorial below, section “Pyneal Toolkit ***–*** Full Pipeline Tutorial”). Users can manually create this configuration file ahead of time, or, if no file exists, the user will be prompted to specify the parameters via the command line when launching. Parameters specified via the command line will be written into the configuration file and saved to disk. Pyneal Scanner will automatically read this configuration file at the start of every scan. Thus, Pyneal Scanner needs to be configured only once at the beginning of each experimental session.

**FIGURE 3:**
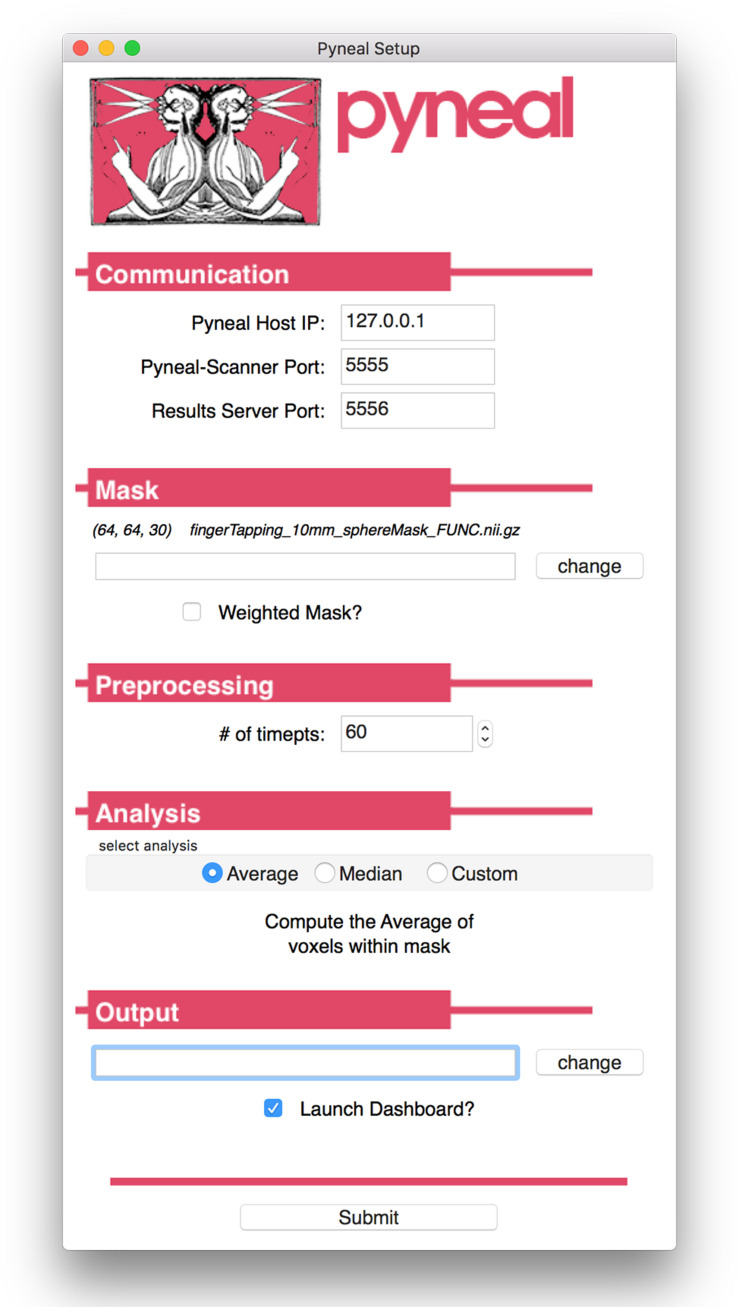
Pyneal Graphical User Interface (GUI). The Pyneal GUI contains the following sections: (1) Communication: allows Pyneal to communicate with Pyneal Scanner and any End Users. This includes the IP address of the computer running Pyneal as well as the port numbers for Pyneal Scanner and End Users to communicate with Pyneal. (2) Mask: users have the option of loading a mask to use during real-time fMRI runs (weighted or unweighted). (3) Preprocessing: users specify the number of timepoints (volumes) in the run. (4) Analysis: users may choose between one of the default options (calculating the average or median of a mask) or importantly can upload a custom analysis script (e.g., correlation between two regions). (5) Output: users specify a location where the output files are saved.

Launching Pyneal is also done via the command line. Upon launching Pyneal at the start of each scan (run), the user is presented with a setup GUI for configuring Pyneal to the current scan (see Figure 3). The setup GUI includes sections for socket communication parameters (e.g., IP address), selecting an input mask, setting preprocessing parameters, choosing analyses, and specifying an output directory. Some parameters, like the socket communication host address and ports, are unlikely to change from experimental session to session, while other parameters, most notably the input mask and output directory, will be specific to experimental session and/or each individual scan. The GUI is populated with the last used settings to minimize set-up time, however, the GUI must be launched before each scan.

The setup GUI asks users to specify the path to an input mask, which will be used during the analysis stage of a scan. If the user selects one of the built-in analysis options (i.e., calculate an *average* or *median*), the mask will define which voxels are included in the calculation. Alternatively, if the user chooses to use a custom analysis, a reference to this mask will be passed into the custom analysis script, which the user is free to use or ignore as needed. In addition, the mask panel also allows users to specify whether or not to use voxel values from the mask as weights in subsequent analyses.

**FIGURE 4:**
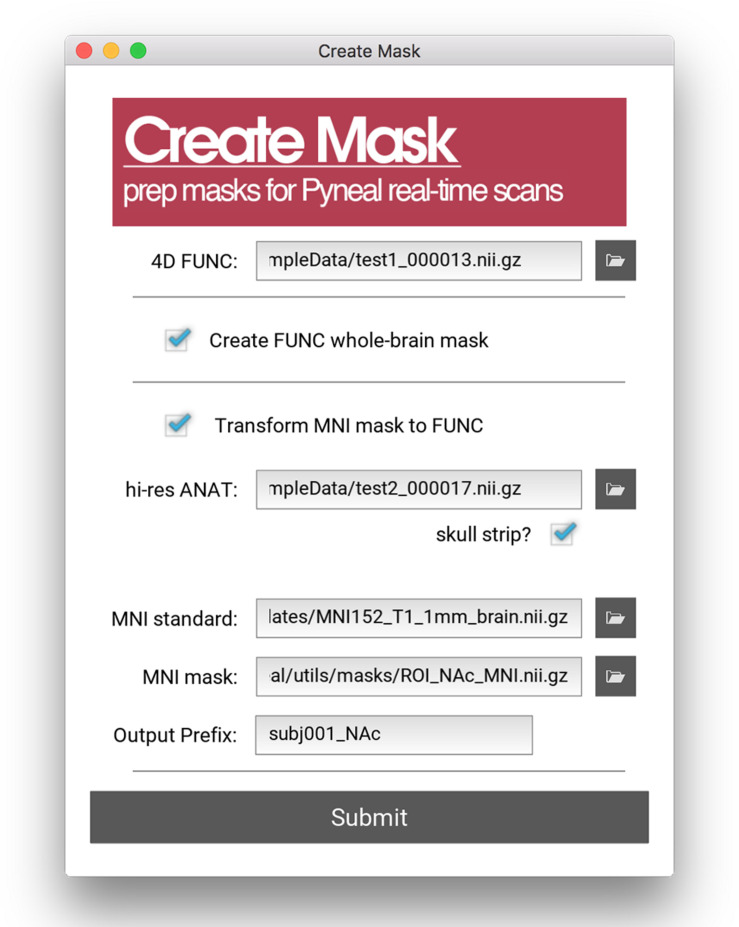
Create Mask GUI. This GUI assists users in making a mask that can be used in analysis during the real-time fMRI runs. Users can choose between making a whole brain mask or a mask from a pre-specified MNI template (e.g., amygdala ROI). Users must load an example functional data file for both mask types. When creating a mask from an MNI template, users must additionally load an anatomical data file, specify the path to the MNI standard brain file, the MNI mask file, and specify the new file name (output prefix). Note, this tool requires FSL.

All analyses in Pyneal take place in the native functional space of the current scan, and as such, this mask is required to match the dimensions and orientation of the incoming functional data. For cases where the user wishes to use an existing anatomical mask in a different imaging space (e.g., MNI space), the Pyneal toolkit includes a Create Mask tool (*utils/createMask.py*) for transforming masks to the functional space of the current subject [see Figure 4; Note that this functionality requires FSL (Jenkinson et al., 2012) to be installed].

Pyneal includes built-in analysis options for calculating the average and median activation levels across all voxels in the supplied mask. For experiments that wish to present neurofeedback from a single ROI, these options may be appropriate. However, one of Pyneal’s primary advantages is the ability to run fully customized analyses. By selecting “custom” in the analysis panel, the user will be prompted to choose a python-based analysis script they have composed. Pyneal requires that a custom script contain certain functions in order to integrate with the rest of the Pyneal pipeline throughout a scan. However, beyond that basic structure, there are few limitations on what users may wish to include. To assist users in designing a custom analysis script, we include a basic template file^6^ with the required named functions and input/output variable names that users can expand upon as needed. The benefit of this approach is that it liberates users to design analysis approaches that are best suited to their experimental questions, all while fully integrating into the existing Pyneal pipeline.

Lastly, users are able to specify an output directory for the current experimental session. During an experimental session, the output from each scan will be saved to its own unique subdirectory within this output directory. The saved output from each scan includes a log file showing all settings and messages recorded throughout the scan, a JSON file containing all of the computed analysis results, and a 4D NIFTI image containing the functional data as received by Pyneal.

Once the user hits “submit,” Pyneal will establish communication with Pyneal Scanner, launch the results server, and wait for the scan to start and data to appear.

### Web-Based Dashboard

**FIGURE 5:**
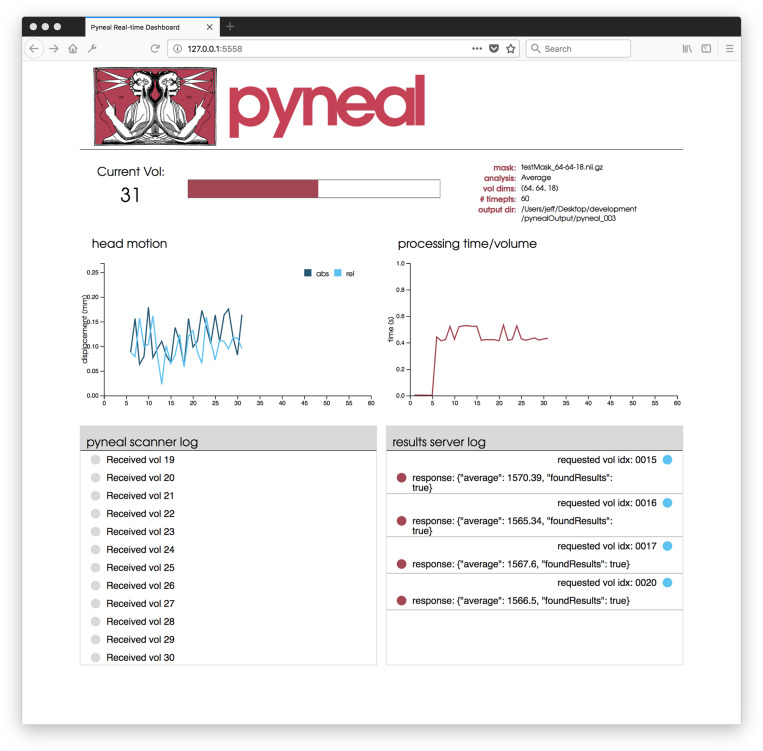
Pyneal Dashboard. This web-based dashboard allows users to monitor analysis and progress during real-time runs. The current volume is displayed along with basic information about the scan (e.g., mask, analysis, etc.). Two plots indicate: (1) head motion (top left) – both relative (compared to previous volume) and absolute (compared to the start of the scan) and (2) processing time for each volume (top right). Two log windows display: (1) messages from Pyneal Scanner (bottom left) and (2) communication between Pyneal and the End User (bottom right).

Once the scan begins, users are presented with a web-based dashboard (see Figure 5) viewable in an internet browser. The dashboard updates in real-time allowing users to view the progress of the scan, and monitor the status via four separate components. A plot in the top-left displays on-going head motion estimates expressed in millimeters relative to both a fixed reference volume (absolute displacement) and the previous volume (relative displacement). In the top-right, a separate plot shows the processing time for each volume. By monitoring this plot, users can ensure that all analyses are completing at a rate that keeps pace with data acquisition. At the bottom, two log windows allow users to watch incoming messages from Pyneal Scanner (bottom left) and communication between Pyneal’s results server and any End User (bottom right).

## Results

Here we present two complementary tutorials and results using real fMRI data. Section “Pyneal Toolkit – Full Pipeline Tutorial” details how to set up and use the Pyneal toolkit. It demonstrates the full pipeline of data flow throughout the Pyneal Toolkit. Section “Pyneal Analysis Tutorial” describes in more detail how to run two example analyses in Pyneal – one using the default built-in ROI-averaging tool in the toolkit and the second using a custom analysis script. Please see: https://github.com/jeffmacinnes/pyneal-tutorial for full access to the data and scripts for both tutorials.

Both tutorials assume the user has downloaded and installed the Pyneal toolkit and the Pyneal Tutorial repositories in their local folder. If so, the following directories should be located in the user’s home directory:

∼/pyneal
∼/pyneal-tutorial

### Pyneal Toolkit – Full Pipeline Tutorial

The goal of this tutorial is to test the Pyneal toolkit’s complete pipeline using conditions similar to what is available at the three major scanner manufacturers. This tutorial uses the Scanner Simulator command line tool that comes with the Pyneal toolkit. This tool mimics the behavior of an actual scanner by writing image data to an output directory at a steady rate (directory and rate specified by the user). The source data (included) are actual scan images from GE, Philips, and Siemens scanners. These data are meant to simulate the format and directory structure typical of each of these platforms. This tutorial allows users to test the complete Pyneal toolkit’s pipeline on any of these platforms prior to actual data collection.

Regardless of scanner type, each platform follows the same general steps:

• Set up the Scan Simulator.
• Set up Pyneal Scanner.
• Set up Pyneal.

Below we provide a complete example using the Siemens’ scanner setup. Please see https://github.com/jeffmacinnes/pyneal-tutorial for source data and information for all scanner types, including examples using GE and Philips scanners.

*Siemens Full Pipeline Tutorial*:

Inside the Siemens_demo folder, there is a directory named scanner. This directory serves as the mock scanner for this tutorial, and follows a structure similar to what is observed on actual Siemens scanners. There’s a single session directory (data) that contains all of the dicom files for two functional series (000013, 000015) and an anatomical series (for more source data detail, see Appendix: Siemens source data within: https://github.com/jeffmacinnes/pyneal-tutorial/blob/master/FullPipelineTutorial.md).

We will use the Scanner Simulator tool to simulate a new functional series, using 000013 as our source data. The new series will appear in the session directory alongside the existing series files, and dicom files will contain the series name 000014.

To perform this tutorial the following steps are required:

I. Launch Siemens_sim.py with the desired input data
• Open a new terminal window and navigate to the Scanner Simulator tool: cd ∼ /pyneal/pyneal_ scanner/simulation/scannerSimulators
• launch Siemens_sim.py, specifying paths to the source directory (∼/pyneal-tutorial/Siemens_ demo/scanner/data) and series numbers (000013). The user can also specify the new series number (-n 000014), and TR (-t 1000) if desired.
python Siemens_sim.py ∼/pyneal- tutorial/Siemens_demo/scanner/data 000013 -t 1000 -n 000014

The user should see details about the current scan, and an option to press ENTER to begin the scan:

————————
Source dir: ∼/pyneal-tutorial/Siemens_ demo/scanner/data
Total Mosaics Found: 60
TR: 1000
Press ENTER to begin the “scan”

Before starting the simulator, first complete the following two steps: setting up Pyneal Scanner and Pyneal.

II. Configure Pyneal Scanner to watch for new scan data in the session directory for the Siemens mock scanner.
• open a second terminal window, and navigate to Pyneal Scanner: cd ∼/pyneal/pyneal_scanner
• create (or edit the existing) scannerConfig.yaml file in this directory to set the scannerMake to Siemens and the scannerBaseDir to the mock scanner folder. The contents of the scannerConfig.yaml file should be:
pynealSocketHost: 127.0.0.1
pynealSocketPort: ‘5555’
scannerBaseDir: ∼/pyneal-tutorial/Siemens_demo/scanner/data
scannerMake: Siemens
• launch Pyneal Scanner:
python pynealScanner.py

The user should see details about the current session, and an indication that Pyneal Scanner is attempting to connect to Pyneal:

================
Session Dir:
∼/pyneal-tutorial/Siemens_demo/scanner/data
Unique Series:
  000013 60 files 1113170 min, 51 s ago
  000015 60 files 1113170 min, 51 s ago
  000017 52 files 1113170 min, 51 s ago
MainThread - Connecting to pynealSocket…

There is nothing more to do in this terminal window. Once Pyneal is set up and the Scan Simulator tool starts, Pyneal Scanner will begin processing new images as they appear and sending the data to Pyneal. The user can monitor the progress via the log messages that appear in this terminal.

III. Configure and launch Pyneal
• Open a third terminal window, navigate to and launch Pyneal.
cd ∼/pyneal
python pyneal.py
• Configure Pyneal for the Siemens tutorial demo:
  ∘ Communication: Set the Pyneal Host IP to 127.0.0.1, the Pyneal-Scanner Port to 5555, and the Results Server Port to 5558.
  ∘ Mask: In the Siemens_demo directory, there is a file named dummyMask_64-64-18.nii.gz. Set the mask value in Pyneal to use this file. This mask was pre-made to match the volume dimensions of the Siemens_demo scan data. This mask is simply a rectangle positioned in the middle slice of the 3D volume, and is for demonstration purposes only. The user can unselect the Weighted Mask? option
  ∘ Preprocessing: Set # of timepts to: 60. The user can keep the Estimate Motion? option selected if preferred.
  ∘ Analysis: Select the Average option.
  ∘ Output: Set the output directory to ∼/ pyneal-tutorial/Siemens_demo/output. Check Launch Dashboard?
• Start Pyneal by pressing Submit.
  ∘ In the Pyneal Scanner terminal, the user will see messages indicating that Pyneal Scanner has successfully set up a connection to Pyneal and that it is waiting for a new seriesDir (which will be created once the scan starts).
  ∘ In addition, the user can open a browser window and enter 127.0.0.1:5558 in the URL bar to see the Pyneal dashboard.

IV. Start demo
• In the first terminal window, where the Scan Simulator tool is running, press ENTER to begin the scan.
• As the scan is progressing, each of the three terminal windows will update with new log messages. In addition, the user can monitor the progress from the dashboard in a web browser at 127.0.0.1:5558.
• As soon at the scan finishes, the user can find the Pyneal output at ∼/pyneal-tutorial/Siemens_ demo/output/pyneal_001. This directory will have:
  ∘ *pynealLog.log:* log file from the current scan.
  ∘ *receivedFunc.nii.gz:* 4D nifti file of the data, as received by Pyneal ^∗^ *results.json:* JSON file containing the analysis results from the current scan.

### Pyneal Analysis Tutorial

The goal of this tutorial is to guide users through two different analyses using Pyneal. We provide real fMRI data (note – this tool also allows for use of randomly generated data). This tutorial uses the pynealScanner_sim.py command line tool that comes with the Pyneal toolkit. This tool takes real or generated data, breaks it apart, and sends it to Pyneal for analysis. The source data (included) is a nifti file from one run of a hand squeezing task. It alternates between blocks of squeeze and rest (each 20 s, repeated five times). The first analysis demonstrates Pyneal’s built-in ROI neurofeedback tool. The second demonstrates use of a custom analysis script: correlating the activation of two ROIs and using it for neurofeedback.

#### Neurofeedback: Single ROI Averaging Using Built-in Analysis Functions

Example: A researcher wishes to provide participants with neurofeedback from the primary motor cortex (M1) in a hand-squeezing task. The M1 ROI is defined on the basis of an anatomical mask using the Juelich atlas in FSL.

*Tutorial*: Ordinarily, the first step is to create a unique mask in functional space of the target ROI (M1). For the purposes of this tutorial, we provide the ROI in subject-specific space for users. We used the left M1 ROI from the Juelich atlas freely available in FSL. We thresholded the mask at 10% and binarized it using fslmaths. Then using flirt, we converted the left M1 mask (in MNI space) to functional space (subject-specific). The resulting mask, L_MotorCortex.nii.gz is now ready to use in this tutorial.

This tutorial uses the Pyneal Scanner simulation script, which is located in:

∼/pyneal/utils/simulation/pyneal Scanner_sim.py

Usage includes:

python pynealScanner_sim.py [–filePath] [–random] [–dims] [–TR] [–sockethost] [–socketport]

Input arguments:

• -f/–filePath: path to 4D nifti image the user wants to use as the “scan” data. Here we are using “func.nii.gz” provided in ∼/pyneal-tutorial/analysis Tutorial as our input data.
• -r/–random: flag to generate random data instead of using a pre-existing nifti image
• -d/–dims: desired dimensions of randomly generated dataset [default: 64 64 18 60]
• -t/–TR: set the TR in ms [default: 1000]
• -sh/–sockethost: IP address Pyneal host [default: 127.0.0.1]
• -sp/–socketport: port number to send 3D volumes over to Pyneal [default: 5555]

To run the tutorial, the following steps are required:

I. Launch pynealScanner_sim.py script

python pynealScanner_sim.py -f ∼/pyneal -tutorial/analysisTutorial/func. nii.gz -t 1000 -sh 127.0.0.1 -sp 5555

Here we are setting the TR to 1000 ms, the host socket number to 127.0.0.1 and the port number to 5555. This tool will simulate the behavior of Pyneal Scanner. During a real scan, Pyneal Scanner will send data to Pyneal over a socket connection. Each transmission comes in two phases: (1) a json header with metadata about the volume and (2) the volume itself.

Once the user hits enter, she should see the following:

Prepping dataset: ∼/pyneal-tutorial/analysisTutorial/func.nii.gz
Dimensions: (64, 64, 18, 208)
TR: 1000
Connecting to Pyneal at 127.0.0.1:5555
waiting for connection…

II. Launch Pyneal using the appropriate configurations. In a new terminal window type:

cd ∼/pyneal
python pyneal.py

This will launch the Pyneal GUI. Configure Pyneal with the following:

∘ Communication: Set the Pyneal Host IP to 127.0.0.1, the Pyneal-Scanner Port to 5555, and the Results Server Port to 5558.
∘ Mask: In the ∼/pyneal-tutorial/analysis Tutorial/masks/ directory, there is a file named L_MotorCortex.nii.gz. Set the mask value in Pyneal to use this file. The user can unselect the Weighted Mask? option. Once the mask is loaded, the GUI should display the volume dimensions of the selected mask (here 64, 64, 18), allowing us to confirm a match with the dimensions of the upcoming scan.
∘ Preprocessing: Set # of timepts to: 208. The user can keep the Estimate Motion? option selected if preferred.
∘ Analysis: Select the Average option.
∘ Output: Set the output directory to ∼/pyneal-tutorial/analysisTutorial/output. Check Launch Dashboard?

The user can then hit Submit to start Pyneal.

III. Start the scan

Back in the Scan Simulator terminal, the user should see a successful connection to Pyneal

connected to pyneal
Press ENTER to begin the “scan”

IV. Hit Enter to begin the simulated scan

As soon as the scan simulation begins, Pyneal Scanner begins processing and transmitting volumes of the provided data (func.nii.gz) to Pyneal, which calculates the mean activation within the target region on each volume and stores the results on the Pyneal’s Results Server. As the scan is progressing, the user should see information about each volume appear in both the Scan Simulator and Pyneal terminals, indicating the volumes are being successfully transmitted and processed.

V. Results
• At the completion of the scan, the user can find the following Pyneal output files in ∼/pyneal- tutorial/analysisTutorial/output/pyneal_ 001 (*Note*: the directory names increase in sequence. If this is the first time saving output to this directory, it will be _001, otherwise it will be a larger number):
  ∘ pynealLog.log: complete log file from the scan.
  ∘ receivedFunc.nii.gz: 4D Nifti of the data, as received by Pyneal.
  ∘ results.json: JSON-formatted file containing the computed analysis results at each timepoint.
• Since the input data here came from a simple hand squeezing task where we computed the average signal within the Left Motor Cortex, we expect to see a fairly robust signal in the results, following the alternating blocks design of the task.
  ∘ To confirm, the user can open the results.json file and plot the results at each timepoint using the user’s preferred tools (e.g., Python, Matlab).

Note – it is also possible to use this setup to test communication with an End User (e.g., experimental presentation script) if desired. See https://jeffmacinnes.github.io/pyneal-docs/simulations/ for more details.

See *MacInnes et al. (2016) for an example of a single ROI analysis using built-in tools in the Pyneal toolkit. For additional examples of rt-fMRI single-ROI neurofeedback studies, see deCharms et al. (2005), Caria et al. (2010), Sulzer et al. (2013b), Greer et al. (2014)*, *Young et al. (2017)*.

#### Neurofeedback: Correlation Between Two ROIs Using a Custom Analysis Script

*Example*: Using a custom analysis script to calculate the correlation between two ROIs and use the correlation as feedback during a task. *E.g., A researcher wishes to calculate the correlation between the primary motor cortex and the caudate nucleus and use that correlated signal as neurofeedback in a hand squeezing task.*

This tutorial uses the Pyneal Scanner simulation script, which is located in:

∼/pyneal/utils/simulation/pyneal Scanner_sim.py

To perform this tutorial the following steps are required:

I. Setup Scan Simulator

Like in the example in “Neurofeedback: Single ROI Averaging Using Built-in Analysis Functions,” the first step is to set up Pyneal Scanner Simulator, which will send our sample dataset to Pyneal for analysis.

Open a new terminal and navigate to the Simulation Tools directory: cd ∼/pyneal/utils/simulation

Run pynealScanner_sim.py and pass in the path to our sample dataset.

Type:
python pynealScanner_sim.py -f ∼/ pyneal-tutorial/analysisTutorial/func. nii.gz -t 1000 -sh 127.0.0.1 -sp 5555

Hit enter. The user should see the simulator prepare the data and wait for a connection to Pyneal:
Prepping dataset: ∼/pyneal-tutorial/
analysisTutorial/func.nii.gz
Dimensions: (64, 64, 18, 208)
TR: 1000
Connecting to Pyneal at 127.0.0.1:5555
waiting for connection…

II. Setup Custom Analysis Script

This tutorial includes a custom analysis script that the user will load into pyneal. This script can be found at: ∼/pyneal-tutorial/analysisTutorial/custom Analysis_ROI_corr.py. Open this file to follow along below. This script is adapted from the customAnalysis Template.py that is included in the Pyneal toolkit.

There are two relevant sections to this script:

**initialize**

The analysis script includes an __init__ method that runs once Pyneal is launched. This section should be used to load any required files and initialize any variables needed once the scan begins.

In the __init__ method in the tutorial script, the user will find the following code block:

## Load the mask files for the 2 ROIs we will compute the correlation between
# Note: we will be ignoring the mask that is passed in from the Pyneal GUI
mask1_path = join(self.customAnalysis Dir, ‘masks/L_Caudate.nii.gz’)
mask2_path = join(self.customAnalysis Dir, ‘masks/L_MotorCortex.nii.gz’)
mask1_img = nib.load(mask1_path)
mask2_img = nib.load(mask2_path)
self.masks = {
‘mask1’: {
‘mask’: mask1_img.get_data() > 0,
# creat boolean mask
‘vals’: np.zeros(self.numTimepts)
# init array to store mean signal
on each timept
},
‘mask2’: {
‘mask’: mask2_img.get_data() > 0,
‘vals’: np.zeros(self.numTimepts)
}
}
## Correlation config
self.corr_window = 10 # number of timepts to calculate correlation over

The above block of code does the following:

• Loads each mask file. Note that while the template provides a reference to the mask file loaded via the Pyneal GUI, we are ignoring that mask and instead loading each mask manually.
• Pre-allocates an array for each mask where we will store the mean signal within that mask on each timepoint.
• Sets the correlation window to 10 timepoints, meaning that, with each new volume that arrives, the correlation between the two ROIs will be computed over the previous 10 timepoints.

**compute**

The compute method will be executed on each incoming volume throughout the scan, and provides the image data (vol) and volume index (volIdx) as inputs. This method should be used to define analysis steps.

In the compute method in the tutorial script, the user will find the following code block:

## Get the mean signal within each mask at this timept
for roi in self.masks:
mask = self.masks[roi][’mask’]
meanSignal = np.mean(vol[mask])
self.masks[roi][’vals’][volIdx] =
meanSignal
## Once enough timepts have accumulated, start calculating rolling correlation
if volIdx > self.corr_window:
# get the timeseries from each ROI
over the correlation window
roi1_ts = self.masks[’mask1’][’vals’]
[volIdx-self.corr_window:volIdx]
roi2_ts = self.masks[’mask2’][’vals’]
[volIdx-self.corr_window:volIdx]
# compute correlation,
return r-value only
Corr = stats.pearsonr(roi1_ts,
roi2_ts)[0]
else:
corr = None
return {’corr’: corr }

The above block of code does the following:

• Computes the mean signal within each mask at the current timepoint.
• Once enough volumes have arrived, computes the correlation between the two ROIs over the specified correlation window.
• Returns the result of the correlation as a dictionary.

The results of any custom script need to be returned as a dictionary. The Pyneal will integrate these results into the existing pipeline and the results will be available via the Pyneal Results Server (for requests from an End User if desired) in the same manner as with the built-in analysis options.

III. Set up Pyneal

Next, configure Pyneal to use the custom analysis script developed above.

In a new terminal, launch Pyneal:

cd ∼/pyneal
python pyneal.py

• Configure Pyneal:
  ∘ Communication: Set the Pyneal Host IP to 127.0.0.1, Pyneal-Scanner Port to 5555, and the Results Server Port to 5558.
  ∘ Mask: In the ∼/pyneal-tutorial/analysis Tutorial/masks/ directory, select L_Motor Cortext.nii.gz. Note that although the custom analysis script overrides the mask supplied here, a valid mask file is required nonetheless.
  ∘ Preprocessing: Set # of timepts to: 208. The user can keep the Estimate Motion? option selected if preferred.
  ∘ Analysis: Select the Custom option. The user will be presented with a file dialog. Select the custom analysis script at ∼/pyneal-
  tutorial/analysisTutorial/custom Analysis_ROI_corr.py.
  ∘ Output: Set the output directory to ∼/pyneal-tutorial/analysisTutorial/output. Check Launch Dashboard?
• Hit Submit to start Pyneal.

IV. Start the scan

Back in the Scan Simulator terminal, the user should see a successful connection to Pyneal

connected to pyneal
Press ENTER to begin the “scan”

• Hit Enter to begin the simulated scan

As the scan is progressing, the user should see information about each volume appear in both the Scan Simulator and the Pyneal terminals, indicating the volumes are being successfully transmitted and processed.

V. Results

• At the completion of the scan, the user can find the following Pyneal output files in ∼/pyneal- tutorial/analysisTutorial/output/pyneal_ 002 (Note: the directory names increase in sequence. If the user completed the single ROI NF tutorial first, it’ll be _002, otherwise it’ll be a different number):
  ∘ pynealLog.log: complete log file from the scan.
  ∘ receivedFunc.nii.gz: 4D Nifti of the data, as received by the Pyneal.
  ∘ results.json: JSON-formatted file containing the computed analysis results at each timepoint.
• The custom analysis script computed a sliding window correlation between the Left Motor Cortex and the Left Caudate throughout the task.
  ∘ To visualize these results, the user can open the results.json file and plot the results at each timepoint using their preferred tools (e.g., Python, Matlab).

## Discussion

### Advantages of the Pyneal Toolkit rt-fMRI Software

A variety of tools currently exist that support real-time fMRI to varying degrees, including AFNI (Cox and Jesmanowicz, 1995), FIRE (Gembris et al., 2000), scanSTAT (Cohen, 2001), STAR (Magland et al., 2011), FieldTrip toolbox extension (Oostenveld et al., 2011), Turbo-BrainVoyager (Goebel, 2012), FRIEND (Sato et al., 2013), BART (Hellrung et al., 2015), OpenNFT (Koush et al., 2017), and Neu3CA-RT (Heunis et al., 2018). At a time when implementing real-time fMRI meant researchers had to develop custom in-house software solutions, these tools presented a valuable alternative, catalyzing new experiments, and supporting pioneering early research with real-time fMRI. Nevertheless, the existing software options are limited in one or more ways that fundamentally restricts who can use them and where, and what types of experiments they support. Please see Table 1 for a comparison of the Pyneal toolkit to the other main rt-fMRI software packages currently available. For example, some of these tools require users to purchase licensing agreements for the package itself (e.g., Turbo-BrainVoyager), or are designed to work inside of commercial software packages like Matlab^7^. In addition, a number of these tools are designed to only support a particular usage of real-time fMRI, like neurofeedback, while not supporting other uses of rt-fMRI. And lastly, even in cases where the underlying code *is* customizable, it often requires proficiency with advanced computer languages like C++. We built the Pyneal toolkit to directly address these limitations.

**TABLE 1:** Comparison of common, currently available real-time fMRI software packages.

Software	Commercial license required?	Cost	Source code available?	Base software language	Network requirements	Main types of built in analyses	Limitations
**Pyneal**	No	Free	Yes	Python	TCP/IP socket communication	Out of the box ROI neurofeedback analyses; *allows fully custom analysis scripts written by users in Python*	Not compatible with multiband data yet; no online data preprocessing yet; has not been tested on Windows environment (no known incompatibilities)
**AFNI plug_realtime**	No (GNU GPL v2.0 license)	Free	Yes	C	TCP/IP socket communication	Data quality assessment (e.g., motion) and neurofeedback	Limited documentation online; limited built-in functions
**Turbo-Brain Voyager**	Yes	Price varies across countries; see https://www.brainvoyager.com/products/purchase.html for current pricing	Not for main software; may be available for plugins and remote extensions	C++	Depends on the MRI manufacturer. Siemens: TCP/IP connection to the imager and default dicom export to the TBV analysis computer. GE/Philips: supports reading the exported files	Incremental: GLM, ERA, motion correction, spatial smoothing, and drift removal; multi-voxel pattern classification; ICA; ROI NF	Commercial license required. Requires sophisticated software knowledge: custom analyses are allowed via user development of plugins; must be in C++. (Note – TBV provides a network interface build on top of the plugin interface. The network interface is available for all programming languages.)
**FRIEND**	No	Free	Yes	C++	TCP/IP socket communication for communicating with End Users via Friend Engine	Image preprocessing, NF, ROI analysis (PSC), and multivoxel pattern decoding	Requires sophisticated software knowledge: custom plugins must be written in C++. Frontend is fully customizable, but users must be comfortable writing socket protocols.
**OpenNFT**	No (GNU GPL v3.0 license)	Free	Yes	Python and Matlab	TCP/IP socket communication	Built-in key volume and time series preprocessing and processing procedures (e.g., spatial smoothing, high pass filtering, despiking); rt QA; incremental and cummulative GLM; the following types of NF: continuous and intermittent activation-based NF; intermittent effective connectivity NF, continuous classification-based NF.	Requires Matlab license (commercial); tested with Siemens and Philips, but should be compatible with other scanner types. Has not been tested on GE to our knowledge.

*ROI, region of analysis; GLM, general linear model; ERA, event related averaging; ICA, independent component analysis; NF, neurofeedback; IP, internet protocol; TCP, transmission control protocol. Note, in this table we exclude packages that are exclusively quality control (for example see rtQC: https://github.com/rtQC-group/rtQC).*

#### Free and Open-Source

The Pyneal toolkit offers a number of key features that make it an appealing package for existing real-time fMRI practitioners as well as those new to the field. First, in support of the growing movement toward open-science, the Pyneal toolkit is free and open source. It is written entirely in Python (see text footnote 2), and all required dependencies are similarly cost-free and open. We chose to use Python specifically because it is sufficiently powerful to handle the computational demands of fMRI analysis in real-time and the language is comparatively easy for users to read and write, an important consideration when designing a package that encourages customization by researchers. Furthermore, the number of libraries designed to aid scientific computing (e.g., Numpy, Scipy, Scikit-learn), and the large user support community worldwide, have lead Python to surge in popularity among the sciences (see Perez et al., 2011), and neuroscience in particular (see Gleeson et al., 2017 and Muller et al., 2015). The Pyneal toolkit follows style and documentation guidelines of scientific python libraries, and when possible uses the same data formats and image orientation conventions as popular neuroimaging libraries (e.g., NiBabel). Moreover, the source code for the Pyneal toolkit is hosted via a GitHub repository, which ensures users can access the most up-to-date code releases, as well as track modifications and revisions to the codebase across time (Perkel, 2016).

#### Flexibility in Handling Multiple Data Formats and Local Computing Configurations

A second advantage the Pyneal toolkit offers is flexibility in handling multiple different data formats and directory structures. MRI data can be represented via a number of different file formats, depending in part on the particular scanner manufacturer and/or automated processing pipelines that modify data before it gets written to disk. For instance, the scanner may store images using a universal medical imaging standard like DICOM, a more specific neuroimaging standard like Nifti, or a proprietary format like the PAR/REC file convention currently seen with Philips scanners. Moreover, even within a given file format, there is considerable variation in *how* data are represented. For instance, a single DICOM image file may represent a 2D slice (GE scanners) or a 3D volume arranged as a 2D mosaic grid (Siemens scanners). Lastly, even when two imaging centers have the same scanners and use the same data formats, there can be differences in how the local computing networks are configured. This affects where data is saved, and how the Pyneal toolkit can access existing pipelines. The Pyneal toolkit was designed to be robust to these differences across scanning environments.

Relatedly, a third advantage is the ability of the Pyneal toolkit to accommodate multiple different environmental variations. Importantly, the Pyneal toolkit splits data handling from real-time analysis tasks into modular components that run via independent processes. Pyneal Scanner is responsible for reading incoming MRI data in whatever form it takes, accessing the raw data, and reformatting to a standardized form that is compatible with subsequent analysis stages of Pyneal. The re-formatted data is then passed to the preprocessing and analysis stage of Pyneal via TCP/IP based interprocess communications. The modular nature of this configuration offers important advantages. For one, Pyneal Scanner and Pyneal are able (though not required) to run on separate workstations. This is important as researchers may lack the administrative permissions needed to significantly modify the computing environment of a shared scanning suite. For example, in a situation where the scanner console does not export images to a shared network directory, Pyneal Scanner can run on the scanner console and pass data to a remote workstation running Pyneal, minimizing the risk of interfering with normal scanner operations. In other situations where the scanner *does* export images to a shared network directory, Pyneal Scanner and Pyneal can run on the same workstation.

The modular nature of the Pyneal toolkit’s design means that it can be modified to support new data formats in the future without having to drastically alter the core codebase. Importantly, if the Pyneal toolkit does not currently support a desired data format, researchers can modify Pyneal Scanner to accommodate their needs without having to modify the rest of the Pyneal toolkit core utilities. As the entire toolkit is free and open-source, users and welcome and encouraged to do so.

#### Fully Customizable Analyses

A fourth, and chief, advantage that the Pyneal toolkit offers is flexibility of analyses. The ability to design and implement uniquely tailored analysis routines via custom analysis scripts means that users can adapt the method to their research question rather than having to constrain their research questions based on the methodology. This flexibility means that the Pyneal toolkit can be used to accommodate a broader and more diverse spectrum of research and experimental goals, offering numerous benefits to the real-time neuroimaging community and general scientific advancement. Importantly, in the Pyneal toolkit, the entire incoming data stream is made available, and by using custom analysis scripts, researchers can extract, manipulate and interrogate whichever portions of that data are most relevant to their question. In addition, researchers are able to use these results in real-time for whatever purpose they choose, including neurofeedback, experimental control, quality-assurance monitoring, etc.

The ability to design and test one’s own analyses will expedite the growth and maturation of real-time neuroimaging more broadly. It is worth highlighting that real-time fMRI is still a comparatively new approach, with many open questions regarding imaging parameters, experimental design, effect sizes, subject populations, long-term outcomes, and general best practices (Sulzer et al., 2013a). Determining satisfactory answers to these questions has been slow, in part due to the limitations of existing software and a small community of users. Customizing analyses in the Pyneal toolkit allows researchers to work in a rapid and iterative way to explore new methods, addressing these questions, and establishing a framework for future studies. It also means that researchers can keep up with the latest analytic advances in their domain without having to rely on external software developers to release new updates for their real-time tools.

In short, the Pyneal toolkit is powerful precisely because it does not presuppose how researchers intend to use it; our conviction is that advances in real-time neuroimaging are best achieved by empowering the community to develop those advances itself.

### Limitations

While the Pyneal toolkit offers a convenient and flexible infrastructure for accessing and using fMRI data in real-time, there are a few limitations with the software presently. First, the Pyneal toolkit does not currently include built-in online denoising of the raw fMRI data. Depending on the application, a user may find that simple denoising steps prior to analysis, such as slow-wave drift removal or head motion correction, may increase the signal-to-noise ratio and improve the statistical power of the analysis. We plan to include built-in options for basic denoising in forthcoming software releases. In the meantime, the current version of the Pyneal toolkit allows users to implement their own denoising steps as part of a customized processing pipeline via a custom analysis script.

Second, the Pyneal toolkit offers built-in support for standard data formats found across the three main scanner manufacturers, but does not currently support multiband acquisitions. As imaging technology advances, multiband acquisitions are becoming increasingly common as a way to increase coverage while maintaining short TRs. As such, we plan to offer built-in multiband support in an upcoming software update. Due to the modular nature of the Pyneal toolkit, multiband support can be integrated as a component of Pyneal Scanner without requiring significant changes to the bulk of the code base.

Third, the Pyneal toolkit was built and tested using Python 3 on Linux and macOS environments. While there are no obvious incompatibilities with a Windows environment, we have not had the resources to thoroughly test the Pyneal toolkit across multiple platforms. We encourage Windows users to run the Pyneal toolkit via a virtual machine configured as a Linux operating system. In future versions of the Pyneal toolkit we hope to offer broader support across platforms, or containerize the application using a tool like Docker^8^ in order to be platform agnostic.

While our team is working to improve the aforementioned limitations, we would also like to extend an invitation to the neuroimaging community to contribute directly to the Pyneal toolkit. The Pyneal toolkit was developed with the open source ethos of sharing and collaboration. It lives in the GitHub ecosystem, which facilitates collaborative work across multiple teams and/or individuals, and offers an easy way for users to submit new features, discuss code modifications in detail, and log bugs as they are discovered. Working collaboratively in this manner ensures efficiency in expanding the software’s capabilities and improving stability. Anyone interested in working on the Pyneal toolkit can find information in the *Contributor Guidelines* and *Contributor Code of Conduct* outlined in the documentation at the Pyneal toolkit GitHub repository at: https://github.com/jeffmacinnes/pyneal.

## Conclusion

In this article we describe the Pyneal toolkit, a free and open-source software platform for rt-fMRI. The Pyneal toolkit provides seamless access to incoming MRI data across a variety of formats, a flexible basis to carry out preprocessing and analysis in real-time, a mechanism to communicate results in real-time with remote devices, and interactive tools to monitor the quality and status of an on-going real-time fMRI experimental session. In addition to a number of basic built-in analysis options, the Pyneal toolkit offers users the flexibility to design and implement fully customized processing pipelines, allowing real-time fMRI analyses to be tailored to the experimental question instead of the other way around [for two examples using the Pyneal toolkit with different experimental approaches see (MacInnes et al., 2016) and (MacDuffie et al., 2018)]. As the rt-fMRI community grows worldwide, new tools are needed that allow researchers to flexibly adapt to suit their unique needs, be that neurofeedback from a single or multiple regions, triggering task flow, or online multivariate classification. The Pyneal toolkit offers researchers a powerful way to address the current open questions in the field, and the flexibility necessary to adapt to answer future questions.

## Data Availability Statement

Pyneal is available at https://github.com/jeffmacinnes/pyneal and full documentation is online at https://jeffmacinnes.github.io/pyneal-docs/.

## Author Contributions

JM designed and developed the software and documentation, and co-wrote the manuscript. KD consulted on software design, provided material support, and co-wrote the manuscript. RA and AS consulted on software design and implementation, provided material support, and revised the manuscript. RR and CP provided material support and revised the manuscript. All authors contributed to the article and approved the submitted version.

## Conflict of Interest

The authors declare that the research was conducted in the absence of any commercial or financial relationships that could be construed as a potential conflict of interest.

## Footnotes

1 Throughout this article we use the term *scan* or *run* to refer to a single, discrete 4D acquisition, and the terms *experimental session* to refer to a collection of scans that are administered to a particular participant in a continuous time window.

2 https://docs.python.org/3.6

3 Throughout this article we will use the phrase “Pyneal toolkit” when referring to the overall toolkit and “Pyneal” when referencing the specific software component comprising Pyneal (as opposed to Pyneal Scanner for example).

4 The Pyneal toolkit documentation, and the figures shown throughout this section, use the terms “Scanner Computer” and “Analysis Computer” to refer to the machines that are running Pyneal Scanner and Pyneal, respectively. However, it is important to note that these terms refer to functional roles: there is no conflict in having the Pyneal Scanner and Pyneal running on the same physical machine.

5 ZeroMQ, a powerful open source library for messaging, is available at https://zeromq.org/ and https://github.com/zeromq.

6 https://github.com/jeffmacinnes/pyneal/blob/master/utils/customAnalyses/customAnalysisTemplate.py

7 https://www.mathworks.com/help/matlab/
